# Comparison of ultrasmall IONPs and Fe salts biocompatibility and activity in multi-cellular in vitro models

**DOI:** 10.1038/s41598-020-72414-8

**Published:** 2020-09-22

**Authors:** Natalia Janik-Olchawa, Agnieszka Drozdz, Damian Ryszawy, Maciej Pudełek, Karolina Planeta, Zuzanna Setkowicz, Maciej Śniegocki, Andrzej Żądło, Beata Ostachowicz, Joanna Chwiej

**Affiliations:** 1 Faculty of Physics and Applied Computer Science, AGH University of Science and Technology, Kraków, Poland; 2 Faculty of Biochemistry Biophysics and Biotechnology, Jagiellonian University, Kraków, Poland; 3 Institute of Zoology and Biomedical Research, Jagiellonian University, Kraków, Poland; 4 Collegium Medicum in Bydgoszcz, Bydgoszcz, Poland

**Keywords:** Biological techniques, Biophysics, Cell biology, Chemical biology, Medical research, Risk factors, Nanoscience and technology

## Abstract

In the paper, the results of the first regular studies of ultra-small iron oxide nanoparticles (IONPs) toxicity in vitro were presented. The influence of PEG-coated NPs with 5 nm magnetite core on six different cell lines was examined. These were: human bronchial fibroblasts, human embryonic kidney cells (HEK293T), two glioblastoma multiforme (GBM) cell lines as well as GBM cells isolated from a brain tumor of patient. Additionally, mouse macrophages were included in the study. The influence of IONPs in three different doses (1, 5 and 25 µg Fe/ml) on the viability, proliferation and migration activity of cells was assessed. Moreover, quantifying the intracellular ROS production, we determined the level of oxidative stress in cells exposed to IONPs. In the paper, for the first time, the effect of Fe in the form of IONPs was compared with the analogical data obtained for iron salts solutions containing the same amount of Fe, on the similar oxidation state. Our results clearly showed that the influence of iron on the living cells strongly depends not only on the used cell line, dose and exposure time but also on the form in which this element was administered to the culture. Notably, nanoparticles can stimulate the proliferation of some cell lines, including glioblastoma multiforme. Compared to Fe salts, they have a stronger negative impact on the viability of the cells tested. Ultra-small NPs, also, more often positively affect cell motility which seem to differ them from the NPs with larger core diameters.

## Introduction

Increasing interest in the application of superparamagnetic iron oxide nanoparticles (IONPs) in various fields of biomedicine, including cancer diagnostics and therapy, entails the necessity of their toxicological profile assessment^1,2^. A first approach to such study falls at the turn of 80s and 90s of the last century when IONPs were introduced to clinics as contrast agents^3,4^. Despite the passage of time, the potentially damaging effects of IONPs still rise a lot of controversies and the adverse effects of both intended and unintended exposure to the nanoparticles (NPs) are still a subject of many scientific research^5–7^.

The in vitro studies constitutes basic method for the assessment of the toxicity and biocompatibility of NPs and other nanomaterials (NMs)^8^. Among their advantages little costs, low complexity of the examined system, short time of the experiment and minimal ethical issues can be included^8,9^. The in vitro assays provide the information on cells life parameters such as viability, proliferation rate, motility or metabolic activity, which are commonly used in the toxicity assessment^9^. Their great advantage is also a possibility to control and reproduce experimental conditions, which significantly increase the repeatability of the obtained results as well as analysis of the biocompatibility and tolerance of higher NPs doses^10–14^. It should be mentioned that in vitro investigations cannot completely replace animal based experiments, but can limit them to the situations when the use of animals is necessary. Moreover, in vitro studies provide valuable mechanistic information on the NPs toxicity after investigation carried in in vivo conditions^15^.

Vakili-Ghartavol et al., and Patil et al., summarized published till now results of the in vitro investigations concerning the toxicity of IONPs, comparing the effects of NPs with different physicochemical properties on various cell lines. In the light of their studies as well as other existing literature, the toxicity of IONPs strongly depends on NPs size, surface coating, dose and the type of cells exposed to their action^10,11,16,17^.

In this paper, the influence of ultra-small IONPs with 5 nm magnetite core and PEG coating (NPs hydrodynamic diameter equal to 47 nm) on different cell lines was examined. Three doses of IONPs, namely 1, 5 and 25 µg Fe/ml, were tested. The span of studied IONPs doses in the literature is very wide. They range from single micrograms of Fe/ml^18–22^ to even milligrams of Fe/ml^23–27^. So, the doses studied by us belong rather to the lower ones analyzed in the context of IONPs toxicity. However, such doses correspond to those used in humans for medical diagnostics (contrast agents in MRI imaging) and those applied in patients for the treatment of iron deficiency anemia^28–30^.

The present study were carried out on six cell lines and their careful choice was dictated by several factors described below. Since respiratory system is the most common path of penetration during the unintended exposure to NMs, human bronchial fibroblasts (NHLF) were analyzed^31–33^. Kidneys constitute the most common removal route for different products of metabolism and foreign bodies^34–36^, including IONPs, therefore human embryonic kidney HEK293T cells were selected for the study as well. To verify theranostic potential of the studied IONPs, their influence on human glioblastoma multiforme (GBM) cells lines U87MG and T98G as well as GBM cells KJT23I that were isolated from brain tumor of patient was examined^37–40^. As NPs administered into circulatory system have been shown to accumulate within macrophages of mononuclear phagocyte system (MPS), present among others in liver and spleen, the mouse macrophages (MAC) were also included in the study^9,36^.

To determine the influence of examined IONPs on cell survival two viability assays, namely MTT and trypan blue tests, were performed. It is necessary to mention that MTT test, evaluating the metabolic activity of cells, is the most commonly used measure of cytotoxicity in case of IONPs^41–44^. Proliferation and migration activity of cells were also assessed in our study. Both parameters are utilized in the assessment of IONPs safety, however, much more rarely, comparing to different viability assays^45–48^. According to the existing literature, one of the mechanisms of IONPs toxicity is oxidative stress^13,49–51^. Therefore, we verified the level of oxidative stress induced by examined IONPs by quantifying and comparison of the intracellular ROS production in IONPs-treated and control cells.

In this paper for the first time (according to our best knowledge), the effect of IONPs on the cells was compared with the appropriate data obtained for iron salts solutions containing the same amount of Fe, on similar oxidation state. Such comparison is necessary to answer the question if iron form is crucial for its influence on the living cells and whether Fe in the form of NPs is more/less safe compared to the free ions of this element.

## Results

### The assessment of the influence on cell proliferation

**Figure 1:**
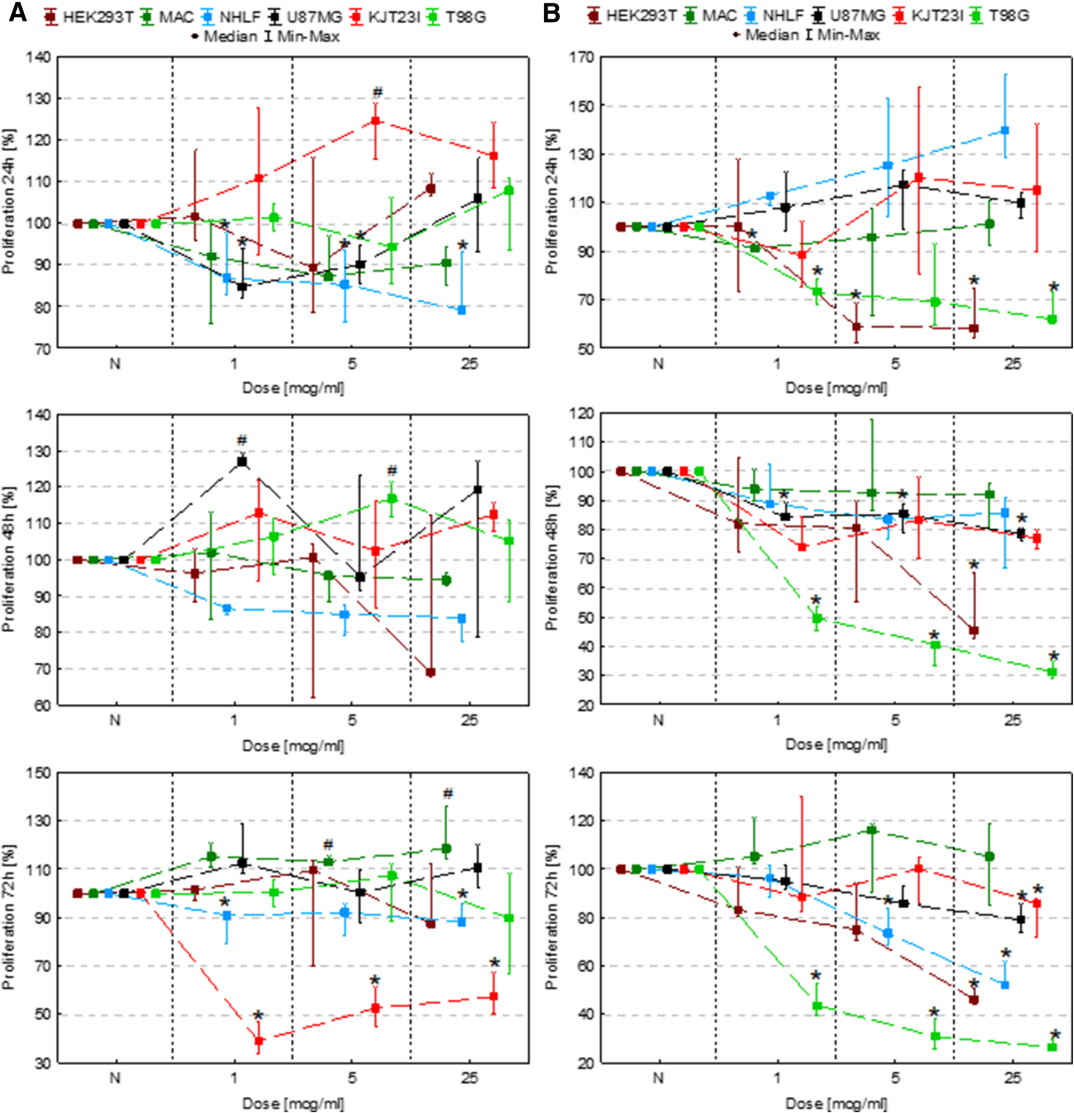
Cell number after 24, 48 and 72 h of exposure to IONPs (**A**) and iron salts solutions (**B**) in the concentrations of 1 µg Fe/ml, 5 µg Fe/ml and 25 µg Fe/ml comparing to the control group (N). The statistically significant (*p* < 0.05) increase in cell line proliferation level compared with corresponding N group was marked with “#” while decrease with “*”. The scales were adjusted for each chart so that data presented are the most legible.

The changes in proliferation level of cells exposed to IONPs and iron salts solutions comparing to control group N are presented as box and whiskers charts in Fig. 1. Based on these experimental data, the growth rates and population doubling times were also calculated for each examined dose of Fe and the obtained results are placed in the Table 1.

**Table 1:** The growth rates and population doubling times for examined cell lines exposed to IONPs and iron salts solutions as well as for corresponding control groups (N).

Cell line	Dose (µg/ml)	IONPs		Fe salts	
		Growth rate^a^ (min^−1^)	Doubling time^b^ (h)	Growth rate (min^−1^)	Doubling time (h)
HEK293T	N	0.00062	18.8	0.00061	18.9
	1	0.00062	18.5	0.00056	20.6
	5	0.00066	17.6	0.00055	21.1
	25	0.00060	19.4	0.00042	27.5
MAC	N	0.00057	20.4	0.00072	16
	1	0.00063	18.4	0.00076	15.1
	5	0.00064	18.1	0.00080	14.5
	25	0.00065	17.7	0.00075	15.4
NHLF	N	0.00023	50.8	0.00020	58.8
	1	0.00021	55.4	0.00018	63.2
	5	0.00021	54.6	0.00011	110
	25	0.00021	56.5	0.00002	664.2
U87MG	N	0.00019	59.8	0.00047	24.6
	1	0.00023	50	0.00046	25.4
	5	0.00020	58.8	0.00042	27.8
	25	0.00022	52.3	0.00040	28.9
KJT23TI	N	0.00053	21.7	0.00026	45.1
	1	0.00026	45.1	0.00023	51.1
	5	0.00032	35.8	0.00025	46.9
	25	0.00035	32.9	0.00021	56.3
T98G	N	0.00041	28.4	0.00041	28
	1	0.00041	28.5	0.00018	63.7
	5	0.00043	27	0.00008	141
	25	0.00037	31.3	0.00004	288.3

^a^The number of doublings occurring per minute.

^b^Calculated as ln(2)/growth rate.

#### Exposure to IONPs

As one can see from Fig. 1A no statistically significant changes in proliferation were observed after 24, 48 and 72 h for HEK293T cells exposed to tested doses of IONPs. The macrophages manifested significantly (*p* < 0.05) increased proliferation after 72 h from administration of 5 µg Fe/ml and 25 µg Fe/ml IONPs solutions. For NHLF cell line the proliferation level diminished significantly after 24-h exposure to all applied doses of IONPs and was lower 72 h from administration of IONPs in concentrations of 1 µg Fe/ml and 25 µg Fe/ml. In case of U87MG cell line, the proliferation activity was significantly lower 24 h after administration of IONPs solutions containing 1 µg Fe/ml and 5 µg Fe/ml. However, after 48-h exposure to IONPs in dose of 1 µg Fe/ml the U87MG proliferation increased. The proliferation level was also elevated in KJT23I cells after 24 h from administration of 5 µg Fe/ml IONPs solution. In turn, after 72 h the KJT23I cells revealed significantly diminished proliferation as a result of the treatment with all IONPs doses used. The T98G cells manifested increased proliferation after 48-h exposure to 5 µg Fe/ml IONPs solution.

#### Exposure to iron salts solutions

After exposition of cells to solutions of Fe salts (Fig. 1B), we observed statistically significant (*p* < 0.05) reduction of proliferation for HEK293T cells (24-h exposure to iron salts in concentrations of 5 µg Fe/ml and 25 µg Fe/ml). In case of HEK293T cells treated with 25 µg Fe/ml the relevantly decreased level of proliferation persisted also after 48 and 72 h of exposition. The macrophages manifested significantly diminished proliferation, only after 24-h and 1 µg Fe/ml dose of iron in the form of salts solution. The proliferation level of NHLF cells was significantly reduced 72 h from the administration of the iron salts solutions in concentrations of 5 µg Fe/ml and 25 µg Fe/ml. The proliferation of U87MG cell line was significantly lower comparing to the control group after 48 h for all iron doses used and after 72 h from administration of solution containing 25 µg Fe/ml. In case of KJT23I cells the proliferation was significantly diminished, only, after 72-h exposure to 25 µg Fe/ml iron salts solution. In turn T98G cell line manifested proliferation decrease after 24 h from administration of solutions containing 1 µg Fe/ml and 25 µg Fe/ml as well as after 48 and 72-h exposure to all applied iron doses.

The analysis of the data presented in Table 1 points that the solutions of Fe salts have negative impact on proliferation of most of the examined cell lines. The effect manifests itself in the increased population doubling time and the decreased growth rate. The IONPs impact on proliferation strongly depends on the type of cells and maybe both negative, as for NHLF and KJT23TI cells and positive, as for macrophages, U87MG and T98G cells presenting decreased population doubling time either for some or all examined doses of IONPs.

### The assessment of the influence on cell viability with trypan blue test

#### Exposure to IONPs

**Figure 2:**
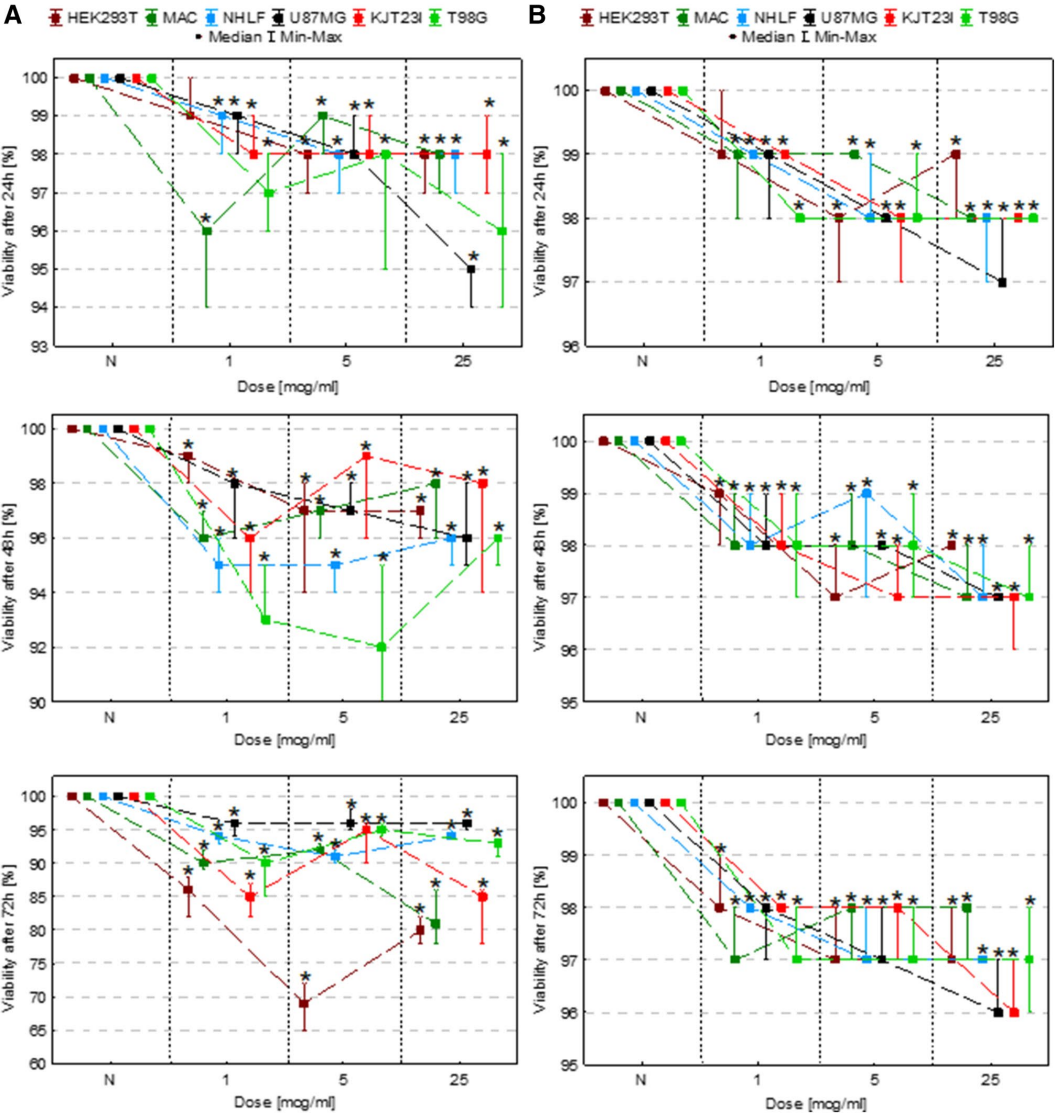
Cell viability after 24, 48 and 72 h of exposure to IONPs (**A**) and iron salts solutions (**B**) in the concentrations of 1 µg Fe/ml, 5 µg Fe/ml and 25 µg Fe/ml comparing to the control group (N). The statistically significant (*p* < 0.05) decreases in cell line viability level compared with corresponding N group were marked with “*”.

The exposure of HEK293T cells to 1 µg Fe/ml IONPs solution resulted in statistically significant (*p* < 0.05) diminishing of their viability after 48 and 72 h. In turn the HEK293T cells treated with 5 µg Fe/ml and 25 µg Fe/ml IONPs solutions manifested diminished viability after 24, 48 and 72 h from administration. The other cell lines (MAC, NHLF, U87MG, KJT23I and T98G) demonstrated decreased viability level after 24, 48 and 72 h for all applied doses of IONPs. The decrease of observed viability is in the 1–5% range after 24-h, 1–8% range after 48-h and 4–30% after 72-h exposures to IONPs (Fig. 2A).

#### Exposure to iron salts solutions

The exposure of HEK293T cells to 1 µg Fe/ml solution of iron salts resulted in statistically significant (*p* < 0.05) reduction of their viability after 48 and 72 h. The HEK293T cells treated with the solutions containing 5 µg Fe/ml and 25 µg Fe/ml manifested diminished viability in all the examined periods of time. The other cell lines (MAC, NHLF, U87MG, KJT23I and T98G) demonstrated decreased viability level after 24, 48 and 72 h for all applied doses of iron. As one can see from the Fig. 2B, the reduction of cells viability reach 1–3% in case of 24 and 48-h exposure and is within the range of 1–4% for 72-h exposure.

### The assessment of the influence on cell metabolic activity with MTT assay

#### Exposure to IONPs

**Figure 3:**
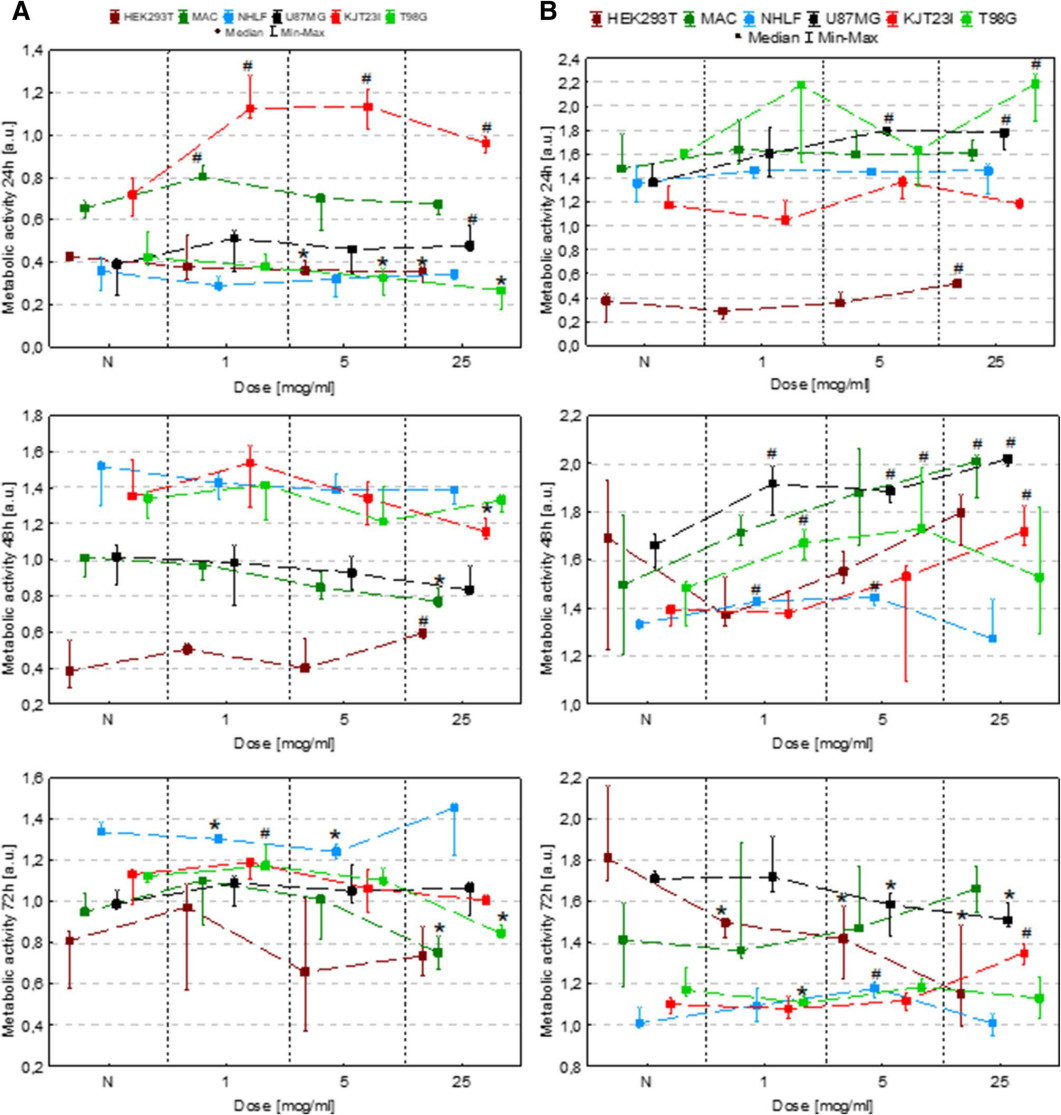
Cell metabolic activity after 24, 48 and 72 h of exposure to IONPs (**A**) and iron salts solutions (**B**) in the concentrations of 1 µg Fe/ml, 5 µg Fe/ml and 25 µg Fe/ml comparing to the control group (N). The statistically significant (*p* < 0.05) increase of the metabolic activity compared with corresponding N group was marked with “#” while decrease with “*”.

In our experiments the metabolic activity of HEK293T cells was significantly diminished (*p* < 0.05) after 24-h exposure to IONPs solutions of 5 µg Fe/ml and 25 µg Fe/ml (Fig. 3A). In turn, in 48th h from the treatment with IONPs solution containing 25 µg Fe/ml, the MTT value for this cell line was elevated. The macrophages revealed increased metabolic activity, only, after 24-h exposure to IONPs in concentration of 1 µg Fe/ml. The significant decrease in MTT values was observed for macrophages treated with IONPs solution containing 25 µg Fe/ml after 48 and 72-h exposure. Metabolic activity of NHLF cells was found to be significantly diminished after 72-h exposure to IONPs solutions of 1 µg Fe/ml and 5 µg Fe/ml. The MTT value for U87MG cell line increased after 24-h treatment with IONPs solution in concentration of 25 µg Fe/ml. In case of KJT23I cells, the metabolic activity was elevated after 24-h exposure to IONPs solutions of 1, 5 and 25 µg Fe/ml. For the cells treated with IONPs in concentration of 25 µg Fe/ml, the MTT value diminished significantly after 48-h exposure. The metabolic activity of T98G cells decreased as a result of 24-h exposure to IONPs solutions of 5 µg Fe/ml and 25 µg Fe/ml and 72-h exposure to the concentration of 25 µg Fe/ml. In turn, after 72-h exposure to IONPs solution containing 1 µg Fe/ml, the MTT value for this cell line was significantly elevated.

#### Exposure to iron salts solutions

Exposition of HEK293T cells to solution of iron salts containing 25 µg Fe/ml (Fig. 3B) revealed their enhanced (*p* < 0.05) metabolic activity after 24-h exposure. In turn, in 72nd hour statistically relevant decrease in their metabolic activity was observed for all the examined doses of Fe. Macrophages exhibited elevated metabolic activity after 48 h following the treatment with the solution containing 25 µg Fe/ml. In case of NHLF cells, increased MTT values were found after 48-h exposure to iron salts solutions in concentrations of 1 µg Fe/ml and 5 µg Fe/ml as well as after 72-h treatment with the dose of 5 µg Fe/ml. The U87MG cells metabolic activity was significantly elevated after 24-h exposure to iron solutions of 5 µg Fe/ml ad 25 µg Fe/ml as well as after 48-h treatment with all the three concentrations of Fe. 72-h exposure of U87MG cells to the doses of 5 µg Fe/ml and 25 µg Fe/ml led to diminished MTT values. KJT23I cells revealed enhanced metabolic activity in 48th and 72nd hour from the treatment with iron salts solution of 25 µg Fe/ml. For T98G cells the MTT values were significantly elevated after 24-h exposure to the dose of 25 µg Fe/ml as well as after 48-h exposure to the doses of 1 µg Fe/ml and 5 µg Fe/ml. In 72nd hour in turn the significant decrease in metabolic activity was found for T98G cells treated with solution of iron salts containing 1 µg Fe/ml.

### The assessment of the influence on cell motility

**Figure 4:**
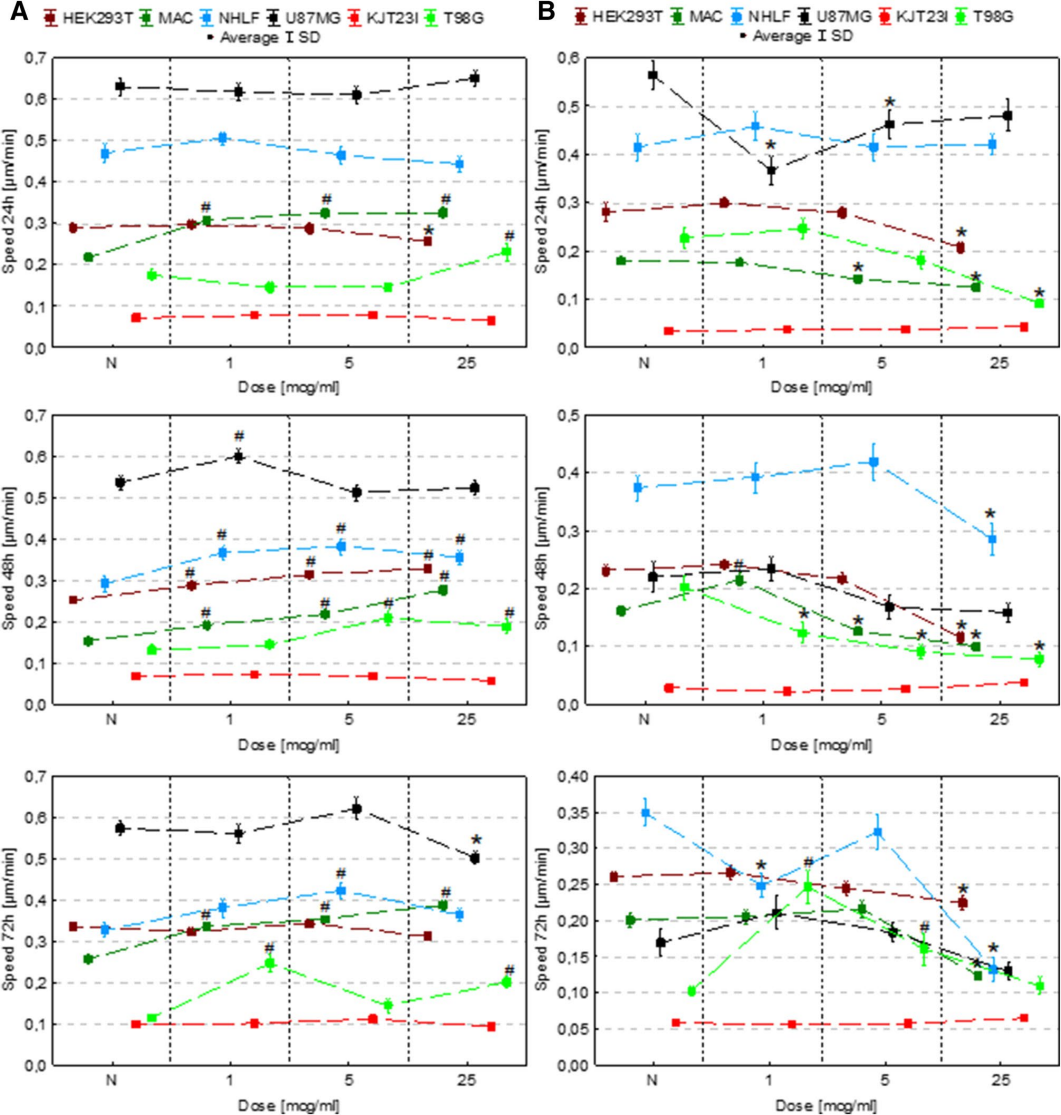
Cell speed after 24, 48 and 72 h of exposure to IONPs (**A**) and iron salts solutions (**B**) in the concentrations of 1 µg Fe/ml, 5 µg Fe/ml and 25 µg Fe/ml comparing to control group (N). The statistically significant (*p* < 0.05) increases in cell line motility level compared with corresponding N group were marked with “#” while decreases with “*”.

**Figure 5:**
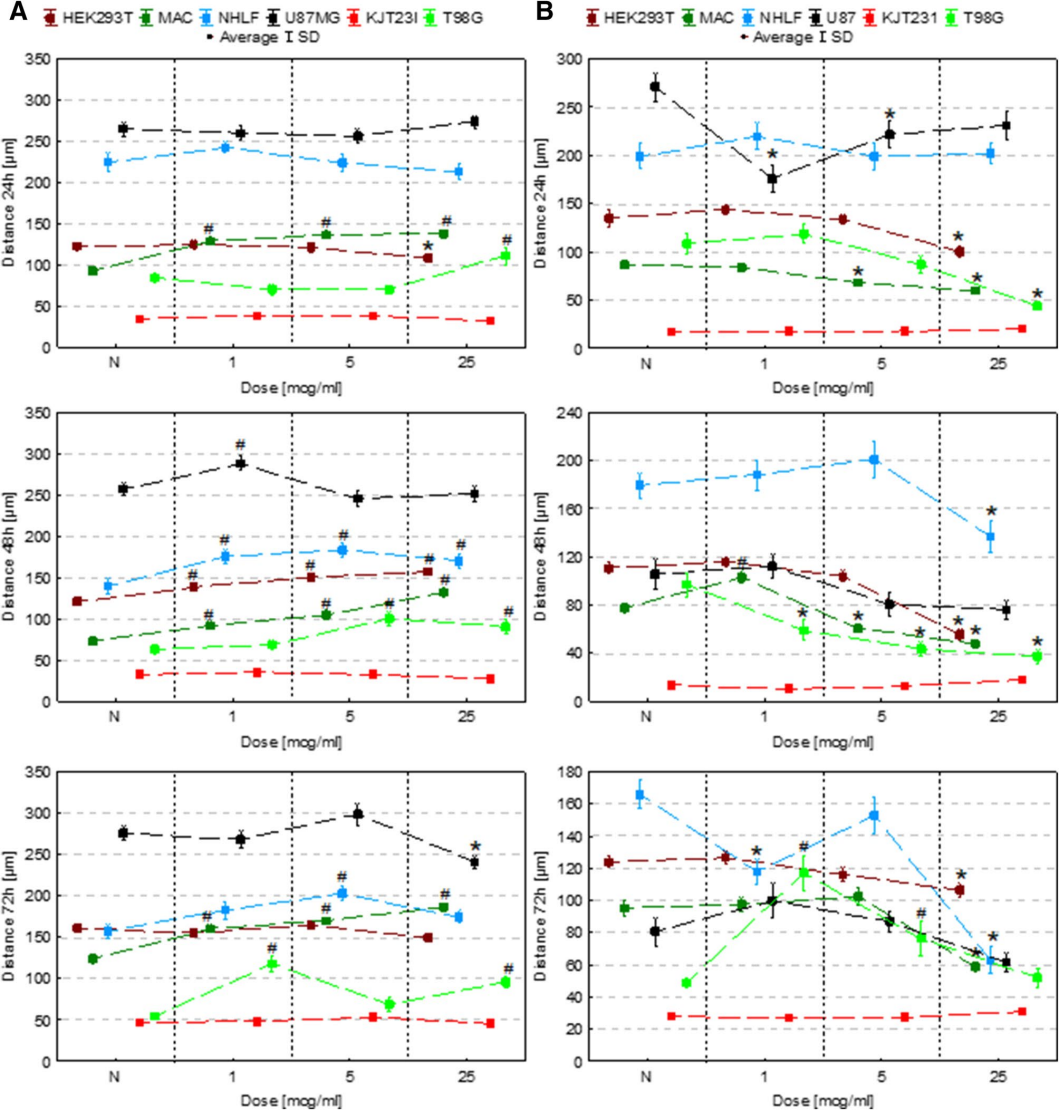
Cell motility (speed and distance travelled) after 24, 48 and 72 h of exposure to iron salts solutions in the concentrations of 1 µg Fe/ml, 5 µg Fe/ml and 25 µg Fe/ml comparing to control group (N). The statistically significant (*p* < 0.05) increases of motility level compared with corresponding N group were marked with “#” while decreases with “*”.

**Figure 6:**
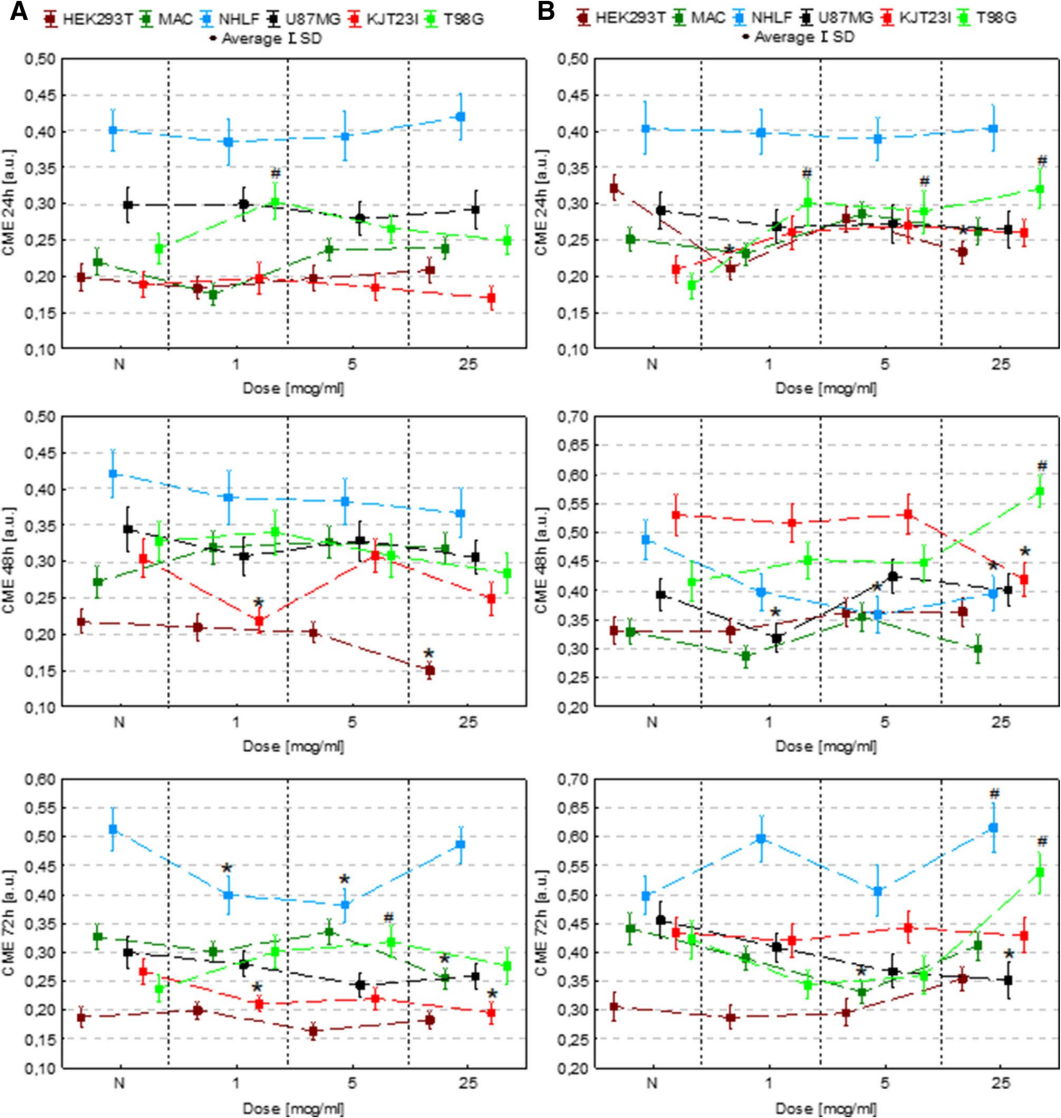
CME coefficient values after 24, 48 and 72 h of exposure to IONPs (**A**) and iron salts solutions (**B**) in the concentrations of 1 µg Fe/ml, 5 µg Fe/ml and 25 µg Fe/ml comparing to control group (N). The statistically significant (*p* < 0.05) increases of motility level compared with corresponding N group were marked with “#” while decreases with “*”.

**Figure 7:**
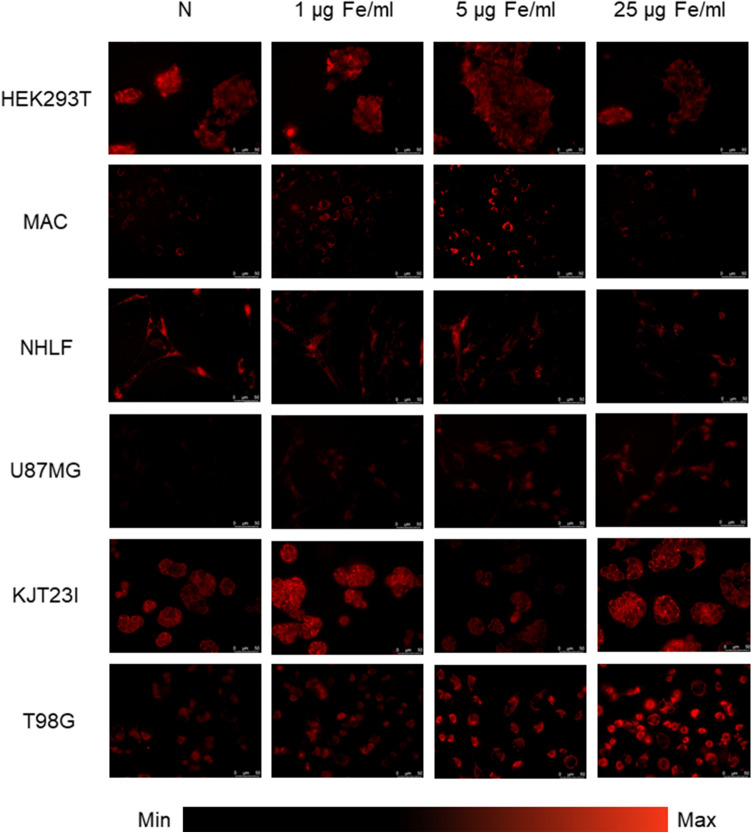
Microscopic images presenting ROS levels after 24 h of exposure to IONPs in the concentrations of 1 µg Fe/ml, 5 µg Fe/ml and 25 µg Fe/ml compared with control group (N).

The motility of cells was evaluated based on their speed and distance travelled (Figs. 4, 5). For this purpose time-lapse video microscopy was applied. To verify if Fe in the form of NPs or salts solutions modify the character of cellular movement, the coefficients of movement efficiency (CME—the ratio of cell displacement to cell trajectory length) were calculated and are presented in Fig. 6. The dot-plots depicting the displacement and the total length of trajectory (distance traveled) for single cells together with the circular plots presenting trajectories of individual cells are, additionally, placed in the Supplementary Information (Figures 2S–7S).

#### Exposure to IONPs

According to the results presented in Figs. 4A and 5A, in 24th hour after the administration of IONPs in dose of 25 µg Fe/ml the HEK293 cells speed and traveled distance decreased significantly (*p* < 0.05). In turn, the 48-h exposure of HEK293T cells to IONPs solutions in concentrations of 1 µg Fe/ml, 5 µg Fe/ml and 25 µg Fe/ml resulted in significant increase of the cellular motility. The macrophages exhibited enhanced motility after 24, 48 as well as 72 h exposure to each of IONPs concentrations tested. The speed and distance travelled by NHLF cells was found to be significantly elevated after 48-h exposure to each of IONPs concentration tested as well as after 72 h from application of IONPs in concentration of 5 µg Fe/ml. The motility of U87MG cells increased after 48-h of the exposure to IONPs solution of 1 µg Fe/ml. In turn, after 72 h from the treatment with the solution of 25 µg Fe/ml, the U87MG cells revealed significantly diminished speed and travelled distance. No statistically significant changes in the motility were found for KJT23I cells. For T98G cells significant increase of speed and distance travelled were observed after 24 h from the treatment with IONPs in concentration of 25 µg Fe/ml. After 48-h exposure to IONPs in concentration of 5 µg Fe/ml and 25 µg Fe/ml as well as after 72-h treatment with the solutions of 1 µg Fe/ml and 25 µg Fe/ml, the motility of T98G cells was also significantly elevated.

#### Exposure to iron salts solutions

In further experiments we investigated the impact of iron salt solutions on cell motile activity (Figs. 4B, 5B). The HEK293T cells exhibited significantly diminished (*p* < 0.05) motility after 24, 48 and 72-h exposure to iron salts solution with the concentration of 25 µg Fe/ml. The distance travelled and speed of macrophages decreased after 24 and 48-h exposure to the solutions of 5 µg Fe/ml and 25 µg Fe/ml as well as after 72-h treatment with iron salts solution of 25 µg Fe/ml. In turn, after 48-h exposure to the solution of 1 µg Fe/ml, the motility of macrophages was significantly elevated. The motility of NHLF cells was diminished after 48 and 72 h treatment with the solution of iron salts of 25 µg Fe/ml as well as after 72-h exposure to solution containing 1 µg Fe/ml. The speed and distance travelled of U87MG cells were found to be significantly decreased in 24th hour of the exposure 1 µg Fe/ml and 5 µg Fe/ml iron doses. For KJT23I cells, significant increase of the motility was observed after 48-h exposure to iron salts solution containing 25 µg Fe/ml. The motility of T98G cells was significantly diminished after 24-h exposure to the solutions containing 25 µg Fe/ml as well as after 48-h treatment with each of the tested iron concentrations. In turn, after 72-h exposure to iron salts in concentration of 1 µg Fe/ml and 5 µg Fe/ml, the speed and distance travelled of T98G cells significantly elevated.

The analysis of the data presented in the Fig. 6 showed that 24-h long incubation of cells with IONPs did not change significantly the value of CME. Longer action of Fe in this form, either left the parameter unchanged or led to the decrease of CME. The iron salts solutions increased the efficiency of movement of T98G cells. What is more, for the highest dose of Fe in this form the effect was noticed for all the examined exposure times. Similar relation was found for NHLF cells after 72-h exposure to Fe salts. Reassuming, Fe in this form, more often than IONPs increased efficiency of cells movement.

### The assessment of the influence on ROS production

**Figure 8:**
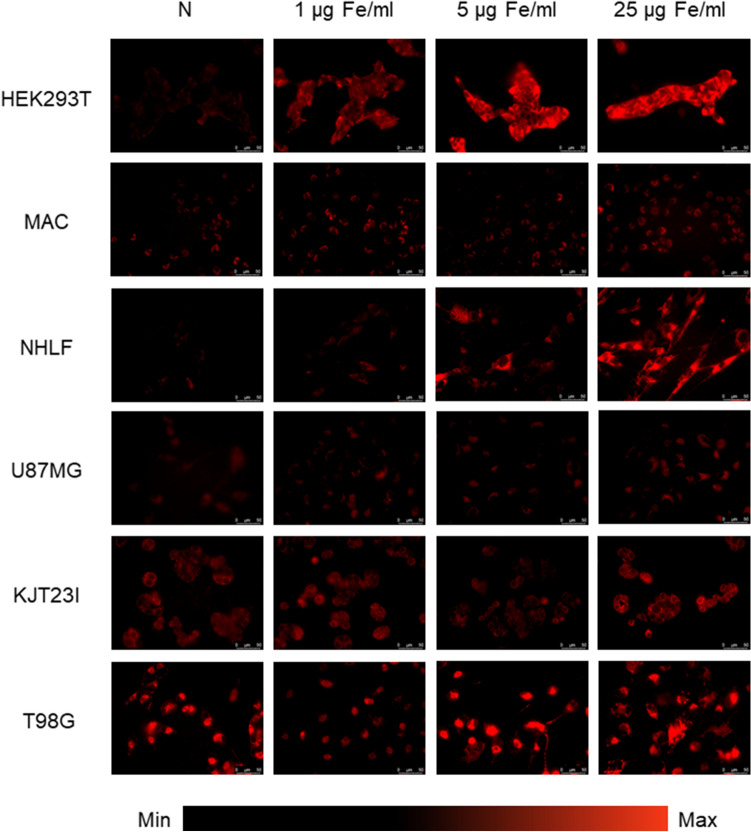
Microscopic images presenting ROS levels after 24 h of exposure to iron salts solutions in the concentrations of 1 µg Fe/ml, 5 µg Fe/ml and 25 µg Fe/ml compared with control group (N).

**Figure 9:**
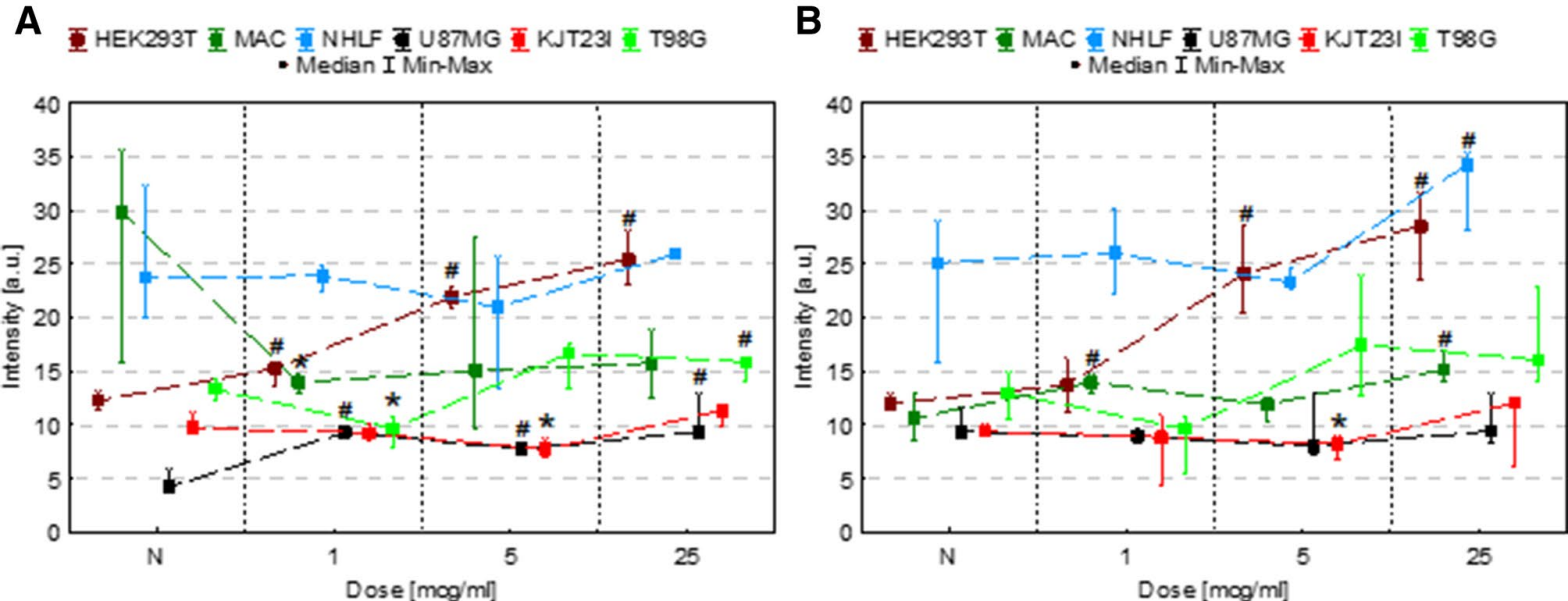
ROS levels after 24 h of exposure to IONPs (**A**) and iron salts solutions (**B**) in the concentrations of 1 µg Fe/ml, 5 µg Fe/ml and 25 µg Fe/ml. The statistically significant (*p* < 0.05) increases in ROS level compared with corresponding N group were marked with “#” while decreases with “*”.

The ROS activity was evaluated by qualitative (Figs. 7, 8) and quantitative (Fig. 9) assessment of the intracellular ROS probe fluorescence intensity. Changes in ROS production were determined by comparison of the results obtained for cells treated with IONPs or iron salts solutions with the ones recorded for corresponding control groups.

#### Exposure to IONPs

The IONPs induced anomalies in ROS production are clearly visible in fluorescence images presented in the Fig. 7 as well as in Fig. 9A containing the results of their quantitative analysis. Apparently, the significantly enhanced (*p* < 0.05) ROS activity was observed for HEK293T and U87MG cells after 24-h exposure to IONPs solutions containing 1 µg Fe/ml, 5 µg Fe/ml and 25 µg Fe/ml, as well as for T98G cells after the treatment with the solution of 25 µg Fe/ml. The significantly diminished level of intracellular ROS was found after 24-h treatment of macrophages and T98G cells with IONPs solution of 1 µg Fe/ml and for KJT23I cells exposed to IONPs in concentration of 5 µg Fe/ml. No statistically significant changes in intracellular ROS level were observed in NHLF cells.

#### Exposure to iron salts solutions

As one can see from the Figs. 8 and 9B, the ROS activity in HEK293T cells was significantly elevated (*p* < 0.05) after 24-h exposure to iron salts solutions with concentrations 5 µg Fe/ml and 25 µg Fe/ml. Enhanced intracellular level of ROS was also found for macrophages after 24-h of the exposure to the solutions containing 1 µg Fe/ml and 25 µg Fe/ml. NHLF cells demonstrated increased ROS activity after 24-h treatment with iron salts solution in concentration of 25 µg Fe/ml. In turn, for KJT23I cells significantly diminished intracellular ROS level was observed after 24-h exposure to the solution of 5 µg Fe/ml. No changes in the ROS activity were observed for U87MG and T98G cell lines treated with the iron salts solutions.

## Discussion

Ultrasmall (with core size less than 10 nm) and ultrafine (with core size less than 5 nm) IONPs seem to be very promising group of IONPs taking into account their possible use in medicine^52^. First, because of superparamagnetic properties of such NPs, they have much less tendency to aggregate and agglomerate^53,54^. They are also able to influence the T1-longitudinal relaxation time of tissues and can serve, in contrary to larger IONPs, as positive contrast agents in MRI^55,56^. Because of smaller core size, their degradation time after internalization into cells of mononuclear phagocyte system is shorter^52^. What is also important from the toxicological point of view, they can be faster excreted from the organism through the renal clearance^57^.

IONPs are generally considered to be biocompatible, however, there is still a lot of controversies on this issue. They result from the fact that the toxicity of NPs very strongly depends on their size, shape, surface properties as well as the used dose^58^. The first step of biocompatibility studies of any newly designed NPs are usually in vitro tests allowing to evaluate the influence of NPs on viability, proliferation, motility or ROS generation^59,60^. In this aspect, a great effort should be done to choose the appropriate cellular lines for investigations, as the answer of different cells to the NPs action may significantly differ^61,62^.

According to our best knowledge, this paper is the first regular in vitro study of the biocompatibility/toxicity of ultra-small IONPs. We examined the impact of NPs with 5 nm magnetite core on six different cellular lines important from the point of view of IONPs metabolism and their theranostic potential. The effect of IONPs on cells viability, proliferation, motility and ROS generation was examined at three doses of NPs and for three exposure times. The analogue set of assays was implemented for the studies on solutions of iron salts containing the same amounts of Fe as in IONPs solutions and the proportions of Fe^2+^ and Fe^3+^ similar as in magnetite. Such an approach can help to answer the question if the form of Fe is crucial for its toxic influence on the studied cell lines.

Our investigation showed that the solutions of iron salts, depending on the used concentration and time of exposure, had a negative impact on cell proliferation. For the longest exposure time and the highest concentration of Fe, proliferation rate diminished for all examined cell lines, except macrophages. Contrary to iron salts, IONPs increased the proliferation rate for some of the examined cells. The effect was mainly observed for human GBM cell lines U87MG and T98G after 48-h treatment with IONPs and in KJT23I cells after 24-h exposure to IONPs. Similar result was found also for macrophages but after 72-h exposure. Thus IONPs, in contrary to Fe in the form of salts solutions may in some cases stimulate the cells proliferation. This observation indicate a potential negative impact of IONPs on the progression of GBM leading to faster tumor growth after MRI related IONPs use. The influence of IONPs on the proliferation of cells is not a commonly used measure of these NMs cytotoxicity. One of the examples of its use is the study of Abakumov MA et al*.*, who verified the influence of IONPs coated with bovine serum albumin (BSA) as well as BSA‐coated and PEGylated on primary human fibroblasts and U251 human glioblastoma cell line. They found that BSA-coated and PEGylated IONPs had the less toxic influence on human fibroblasts and explained this effect by the protective action of PEG‐coating, which prevents the active uptake of NPs and delays their degradation and the release of iron ions^63^. Functionalization of our NPs with PEG might have caused that their negative impact on cell proliferation was usually less than this observed for Fe salts. Such a conclusion, however, is not in agreement with our results concerning the effect of Fe in the two examined forms on cellular viability. Although, both Fe in the form of magnetite nanoparticles and solutions of the salts of the element had negative impact on the viability of cells, the effect was more prominent in case of IONPs and 72-h exposure of some cell lines to IONPs reduced the viability of cells even 20–30 percent comparing to the appropriate controls.

In our study we were focused on the relatively low doses of IONPs, however important from the point of view of NPs applications in humans for medical purposes. The doses varying from 1 to 25 µg Fe/ml correspond to clinical doses of IONPs-based contrast agents (lower) and drugs used for the treatment of iron deficiency anemia (higher)^28,29,64^. According to the most of in vitro studies, for such IONPs doses the lack or low toxicity is observed^13,14,22,23^. However, this is not the rule, as sometimes the cytotoxic effect is observed even at very low doses and short exposition time^20^.

The MTT test of NADH—dependent enzymatic activity is, undoubtedly, the most frequently used assay of IONPs cytotoxicity determination^11,12,16^. The existing literature shows that cytotoxic effect of IONPs on cell metabolic activity depends on the time of exposure and concentration of NPs^13,19^. Our study revealed the lack of the influence of iron salts solutions on the measured with MTT test metabolic activity of macrophages. For all the other cell lines 24 and/or 48-h exposure to Fe in this form led to significant increase of metabolic activity. After the next 24-h of the treatment, the effect persisted only for NHLF and KJT23I cells whilst the metabolic activity of HEK293T, U87MG and T98G cells decreased significantly comparing to the appropriate controls. The effect of IONPs on cell metabolic activity was observed mostly after 24-h exposure. 24-h treatment of U87MG and KJT23I cells as well as macrophages with IONPs increased their metabolic activity. The opposite relation was observed for HEK293T and T98G cells. The changes of metabolic activity were observed also after longer exposure to IONPs, however, the presence of any regularities was not found here. Unlike existing studies^20,23,65^ our research revealed that IONPs may stimulate cellular metabolism and such an effect was noticed after 24-h incubation for U87MG, KJT23I cells and macrophages. Although this observation may suggest a beneficial impact of the NPs on the enzymatic activity of macrophages, in case of tumor cell lines U87MG and KJT23I such an effect seem to be rather undesirable. Our studies also show that results obtained with MTT test do not often correlate with the results of proliferation measured by cell counting. So, using MTT test by many researchers as a proliferation test may lead to false conclusions.

In our studies IONPs, much more often than iron salts solutions, had positive impact on motility of cells which manifests in increased values of their speed and distance travelled. For macrophages and NHLF cells the effect was observed already after 24-h exposure to IONPs. For other examined cells the motility increased, mostly, after 48-h treatment. The exposure of cells to iron in the form of salts of the element usually diminished the motility of the cells in dose-dependent manner and only few exceptions from this rule were found. Therefore, in the light of the obtained results, the influence of Fe on the parameters describing the motility of the cells strongly depend on the Fe form and it is usually positive in case of IONPs and negative in case of the iron salts solutions. These observation, together with the pro-proliferative effect of IONPs on GBM cells, may testify against using IONPs in GBM treatment. The increase of GBM cell motility after IONPs administration suggest the pro-invasive activity of IONPs. Increased GBM cells invasiveness within brain tissue together with faster local tumor mass growth may lead to worse patients survival prognosis.

It should be emphasized that our observation, made for the cell lines treated with the PEG-coated IONPs, is quite unique since most of the papers report the cells motility impairment when exposed to IONPs^45–48^. From the biokinetic point of view the colloidal sizes as well as concentrations of NPs tested in the aforementioned papers are comparable with those used in our investigation^9^. Therefore, the observed discrepancies in the migration rate probably should be assigned to the differences in the core size of the examined IONPs. Moreover, according to our results pointing on the potential of IONPs and Fe salts to generate oxidative stress in dose dependent manner, we hypothesize that the observed modulation of motility within some of examined cell lines may be result of induced disbalance within intracellular redox homeostasis. However, precise evaluation of mechanisms regulating such phenomenon requires much more extensive experimental approach.

The therapy using SPIONs can be carried out by using quite well-known phenomena such as magnetic hyperthermia, magnetic separation or targeted drug delivery. Recently, other possibilities of their application for therapeutic purposes are also being considered and one of them is associated with the exhibited by magnetite NPs, the catalytic activity analogous to peroxidase^66^ . Magnetite, containing the iron on the second and third oxidation state, may be substrates to Fenton’s and Haber–Weiss reactions, respectively. In Haber–Weiss reaction, Fe^3+^ ions are reduced into Fe^2+^ ions which catalyze the Fenton’s reaction, leading to conversion of endogenous H_2_O_2_ into a highly cytotoxic hydroxyl radical that can induce cancer cell death^67–69^. This newly defined chemodynamic therapy is therefore based on the increased production of free radicals formed in the Fenton’s reaction. Therefore, one aspect of our research was evaluation of the impact of examined NPs on the intracellular production of free radicals in GBM cells and the comparison with analogous data obtained for cells important from the point of view of the exposure and metabolism of nanomaterials. IONPs increased ROS production in HEK293T and U87MG cells for all the examined doses of Fe, however, the evident enhancement of the effect together with the used dose of NPs was found only for the first mentioned cellular line. T98G cells presented dose dependent ROS levels—decreased for the lowest IONPs dose and elevated for the highest dose. For macrophages and KJT23I cell line, the level of ROS diminished as a result of exposure to NPs. The effect of Fe on HEK293T and KJT23I cells was almost independent on the form of the element in the solution and, similarly as for IONPs, the solutions of iron salts leaded to increased ROS activity in HEK293T cells and decreased activity in case of KJT23I cells. The opposite relation was found for macrophages for which the exposure to Fe salts solutions increased significantly ROS production. As one can see, the tested cells differ significantly in respect to the susceptibility to the treatment leading to the ROS overproduction. The observed differences, especially in case of tumor cells, may result from the mutations and epigenetic changes leading to the modifications in the expression of enzymes such as catalase or superoxide dismutase and in intracellular glutathione pool which are the main players in the regulation of ROS level. Their higher activity in exposed cells may quickly exclude ROS from the cytoplasm even when their production increase as a result of IONPs or Fe salts action. Different expression patterns of the oxidative stress-regulating enzymes may be one of the reason of diminished level of intracellular ROS in KJT23I cells exposed to IONPs and Fe salts. On the other hand, it is worth emphasizing that KJT23I cells grow in colonies which provide close intercellular communication and the possibility of collective response and attenuation of Fe induced cytotoxicity (by-stander effect)^70^. A quite worrying phenomenon is the strong effect of iron in both forms on ROS production in kidney HEK293T cells. As renal clearance is the main route for removing of nanomaterials and/or the products of their degradation from the body, such negative influence may point at the possibility of the organs damage after the treatment with IONPs. Such a conclusion seems to be in agreement with our previous studies in vivo, which showed that rat exposure to various IONPs may lead to significant elemental and biochemical anomalies within the kidneys^71,72^.

## Conclusions

In this paper the first regular study of the biocompatibility of the ultra-small IONPs under in vitro conditions was presented. The obtained results were compared with data received for the solutions of Fe salts containing Fe^2+^ and Fe^3+^ in the proportions similar to magnetite being the core material of the examined NPs. The obtained results showed that Fe salts in higher doses diminished proliferation rate for most of the examined cell lines which was manifested by the decreased growth rates and increased doubling times of populations. In turn IONPs, for some doses and exposure times increased cell proliferation and this phenomenon was noticed for tumor cell lines (U87MG, T98G and KJT23I) and macrophages. In the light of our findings, both iron salts and IONPs diminished the viability of all examined cells. However, the effect was much more prominent for NPs. Furthermore, Fe in the form of salts solutions did not have any influence on metabolic activity of macrophages but, for some conditions, significantly increased the NADH – dependent enzymatic activity of other cell lines. IONPs influenced the metabolic activity of all examined cells and the effect strongly depended on the experimental conditions. What is more, IONPs very often improved the motility of cells, while the exposure to the Fe in the form of salts solutions usually diminished the speed and distance travelled by cells in time-dependent manner. Finally, both iron salts and IONPs affected the ROS activity of some examined cell lines. The exposure to Fe in the form of salts solutions enhanced ROS production in NHLF and HEK293T cells as well as macrophages but decreased the ROS level of KJT23I cell line. The treatment with IONPs increased ROS activity in HEK293T and U87MG cells, while in macrophages and KJT23I cells the diminished level of ROS was found as a result of their action. In turn, T98G cells exposed to IONPs revealed positive correlation between administered dose of NPs and ROS production. Reassuming, our results clearly suggest that the influence of iron on the living cells strongly depends not only to the used cellular line, dose and exposure time but also on the form in which this element was administered to the culture.

## Materials and methods

### Cell lines

The cell lines selected for the study included primary human bronchial fibroblasts NHLF derived from bronchial biopsy, human embryonic kidney cells HEK293T (ATCC, CRL-3216), human GBM cell lines U87MG (ATCC, HTB-14), T98G (ATCC, CRL-1690) and KJT23I cells isolated from human brain tumor in the Clinic of Neurosurgery and Neurotraumatology of University Hospital in Bydgoszcz according to the consent of the Bioethics Commission for the use of cellular material collected from patients in neurooncological operations (Decision No. KB 535/2017 issued on 13.06.2017 by Bioethical Commission at University of Nicolaus Copernicus in Toruń). Additionally, mouse P388D1 macrophages Mf (ATCC, CCL-46) were the subject of examination.

### IONPs solution

Superparamagnetic iron (II,III) oxide nanoparticles (Sigma-Aldrich, 790508) with PEG coating and concentration of iron equal to 1 mg/ml were dispersed in distilled water. According to the information provided by manufacturer the average nanoparticles size, assessed with transmission electron microscopy (TEM), was 5 nm. The hydrodynamic diameter of the particles as well as their Z-potential were measured with the use of dynamic light scattering (DLS) technique using Malvern Zetasizer Nano ZS instrument at the wavelength of 532 nm. The diameter in the solution was estimated to 47.34 nm with the polydispersity index (PDI) of 0.408. In turn the Z-potential was equal to − 9.8 mV. The volume distribution of the hydrodynamic diameter for the examined nanoparticles was added to the Supplementary materials as a Figure 1S. Initial concentration of Fe in the IONPs solution was strictly determined by TXRF method using Rigaku Nanohunter II spectrometer and was equal to 897 (± 51) ppm. The IONPs stock solution was diluted to final iron concentrations of 1, 5 and 25 µg/ml, that were applied to the selected cell lines.

### Iron salts solution

Iron(III) sulfate hydrate (Sigma-Aldrich, F0638) and iron(II) sulfate heptahydrate (Sigma-Aldrich, F8633) were mixed in a 2:1 molar ratio in distilled water, respectively, to obtained proportions of Fe on the second and third oxidation state similar to those in magnetite. Iron salts solution was administered in concentrations corresponding to those of iron in the applied IONPs solutions, namely 1, 5 and 25 µg/ml.

### Cell culture

Cells were cultured in 25 cm^2^ polystyrene culture flasks (Eppendorf) in DMEM medium with high glucose (4,500 mg/l) (Sigma-Aldrich), supplemented with 10% fetal bovine serum (Gibco) and 1% antibiotics: penicillin/streptomycin cocktail (Sigma-Aldrich). The cultures were grown under strictly controlled conditions: 5% CO_2_, temperature 37 °C. The medium was changed every two days and the cells passaged when the confluence reached 80%. For that purpose the cells were washed with PBS without Ca^2+^/Mg^2+^ (Corning), harvested with 0.25% Trypsin-EDTA solution (Gibco), resuspended in fresh medium, counted using Beckman Z2 particle counter and seeded into plastic culture dishes.

### Cell proliferation and viability assays

Cells were seeded into 12-well culture plate at the density of 2 × 10^4^/cm^2^ and cultivated in DMEM medium. After 24 h, the culture medium was changed to fresh and the IONPs were administered at the three examined concentrations of iron, namely 1, 5 and 25 µg/ml. Culture medium without the IONPs was a control in each experiment. Cell proliferation analysis was performed using a Coulter Z2 Counter. After 24, 48 and 72 h from administration of IONPs, the cells were harvested and counted. In the next step doubling time and grow rate were calculated. In addition, all experiments were carried out for the iron salts solutions containing Fe at the concentration of 1, 5 and 25 µg/ml and in the appropriate proportion of Fe^2+^ and Fe^3+^.

Trypan blue was used for the determination of cells viability. The cells were passaged, centrifuged, resuspended in PBS and mixed with 0.4% trypan blue (Sigma-Aldrich) in ratio 1:1. After 2 min incubation, the cells were examined using a Bürker hemocytometer.

Cell metabolic activity was estimated with MTT assay. The cells were seeded into 96-well culture plate at the density of 5 × 10^3^ per well and cultivated for 24 h before administration of Fe in the form of IONPs or solution of iron salts. After incubation for 24, 48, 72 h from administration of Fe in tested forms, 12 µl of MTT (Sigma-Aldrich) solution was added to each well to a final concentration of 5 mg/ml and incubated at 37 °C for 4 h. The medium was removed carefully and 100 µl DMSO was added to each well and mixed until formazan crystals were dissolved. The absorbance of colored solution was measured using a plate reader (Multiskan FC Microplate Reader; Thermo Fisher Scientific) at the wavelength of 570 nm.

### Cell motility

The cells were seeded into 12-well culture plate at the density of 2 × 10^4^/cm^2^ and incubated in DMEM. After 24 h culture medium was replaced by DMEM with IONPs or solutions of iron salts at the examined concentrations of Fe. Cell movement was registered 24, 48 and 72 h after administration of tested forms of Fe, for 8 h with 5 min intervals using Leica DMI6000B microscope equipped with a IMC contrast optics, DFC360FX CCD camera and chamber allowing the control of CO_2_ level and temperature. Average parameters of cell movement, namely average speed of cell movement (µm/min) and distance (µm) were quantified from the trajectories of 50 cells in each condition using Hiro program written by Czapla as described in the paper of Ryszawy et al.^73^.

### ROS assay

Cells were seeded into 24-well imaging culture plate at the density of 2 × 10^4^/cm^2^ and cultivated for 24 h before administration of IONPs (iron salts solutions). After 24, 48 and 72 h from administration of Fe in the form of IONPs or iron salts solutions, cells were incubated in the presence of CellROX Orange reagent (Invitrogen, C10443) at the concentration of 5 µM for 30 min at 37 °C. Afterwards, the reagent was replaced by FluoroBrite DMEM (Invitrogen, A1896702) containing 10% FBS and 4 mM GlutaMax (Invitrogen, 35050061). Images were acquired with Leica DMI6000B fluorescence microscope and quantitative analysis of intracellular level of ROS was performed with ImageJ software^74,75^.

### Statistical analysis

The Mann–Whitney *U*-test was applied for statistical evaluation of changes in the cells proliferation, viability and ROS activity after exposure to each of the analyzed doses of Fe in the form of IONPs and iron salts. The choice of nonparametric statistical test was dictated by the fact that obtained data might not meet the assumptions concerning the normality, homoscedasticity and linearity which are necessary for use of parametric test. In turn, for assessing the significance of observed changes in cell motility the parametric Student’s *t*-test was applied. The differences between cells exposed to iron salts/IONPs solution and control group were analyzed at the significance level of 5%. The statistical analysis was performed with StatSoft, Inc. (2005). STATISTICA (data analysis software system), version 7.1. www.statsoft.com.

## Supplementary information

Supplementary information.
