# Supplementary material for: Comparison of ultrasmall IONPs and Fe salts biocompatibility and activity in multi-cellular in vitro models
Source: Sci Rep. 2020 Sep 22;10:15447. doi: 10.1038/s41598-020-72414-8 (PMC7508949; doi:10.1038/s41598-020-72414-8)
Supplement: Supplementary file 1 — Supplementary information. [file 41598_2020_72414_MOESM1_ESM.docx]

**Supplementary Information**


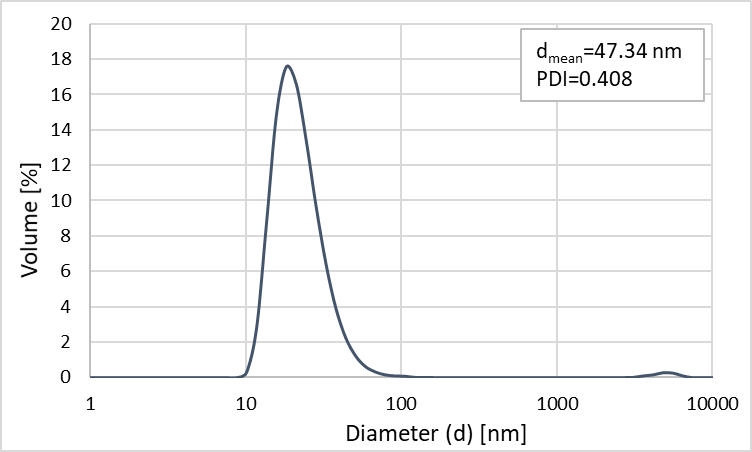
 **
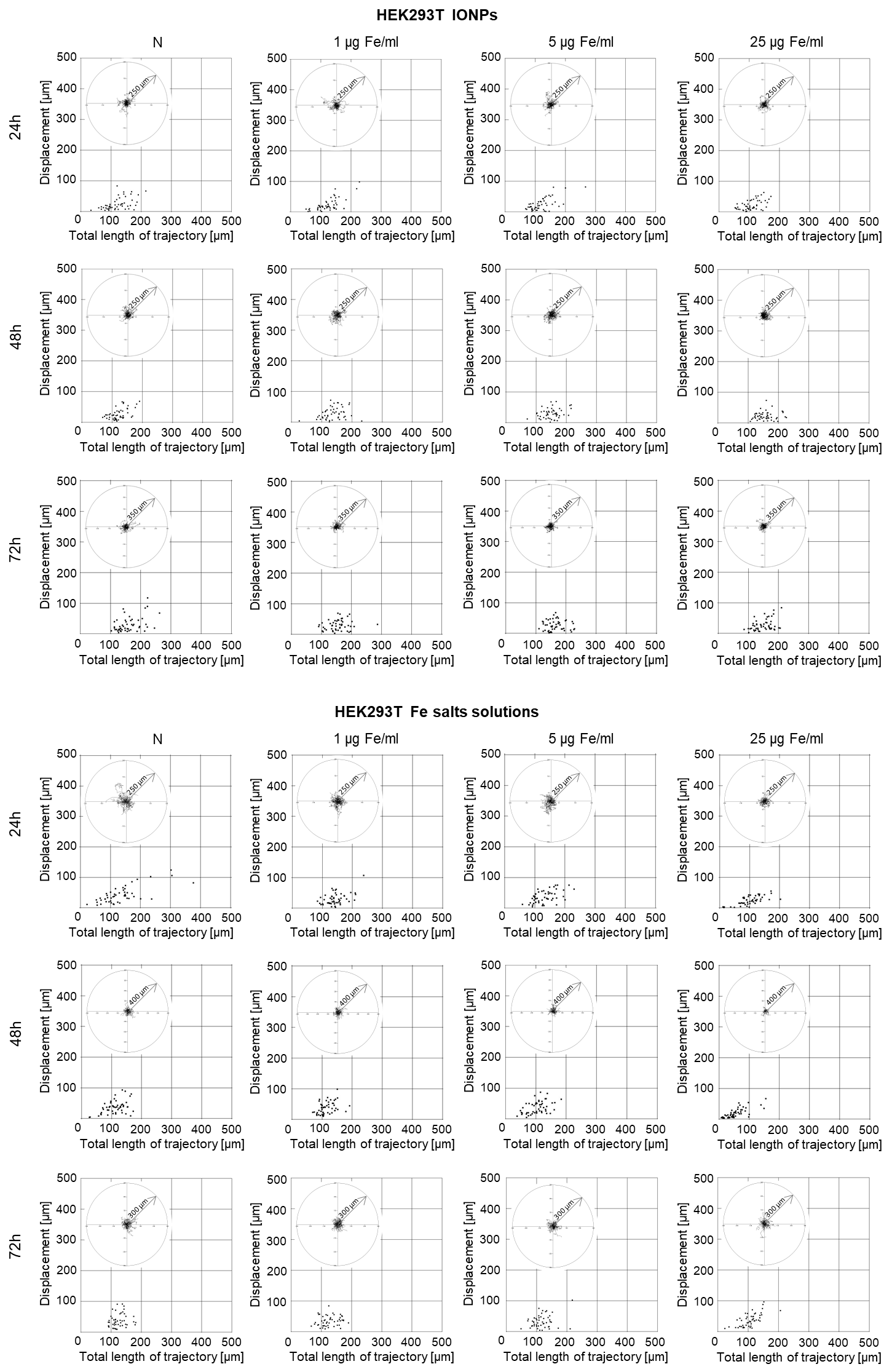
**

Figure 1S. DLS plot showing the size distribution by volume of examined nanoparticles.

Figure 2S. Dot-plots depict displacement and total length of trajectory (distance) calculated for single HEK293T cells exposed to IONPs and iron salts solutions as well as for corresponding control groups N. Circular plots present trajectories of individual cells.


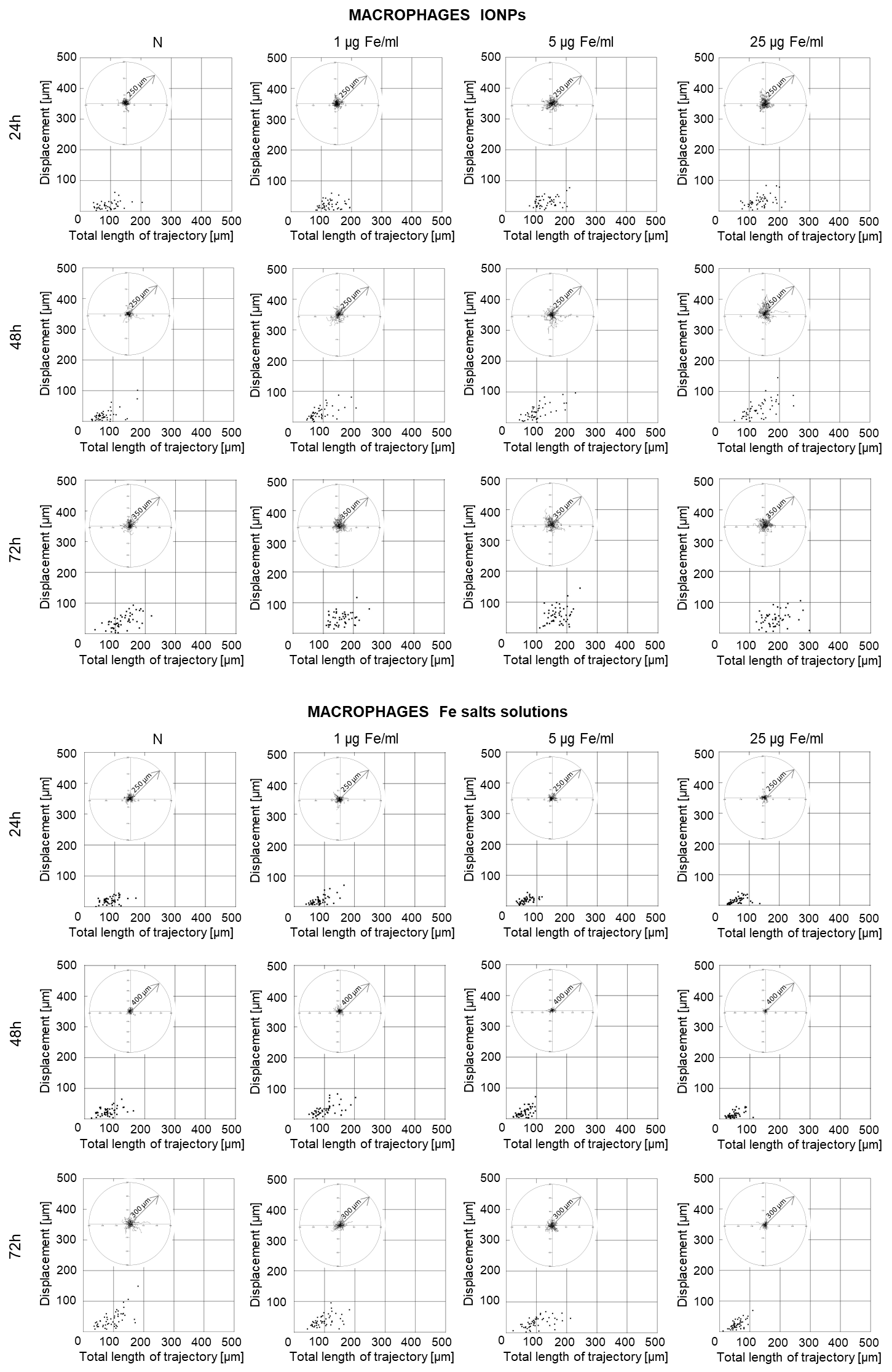

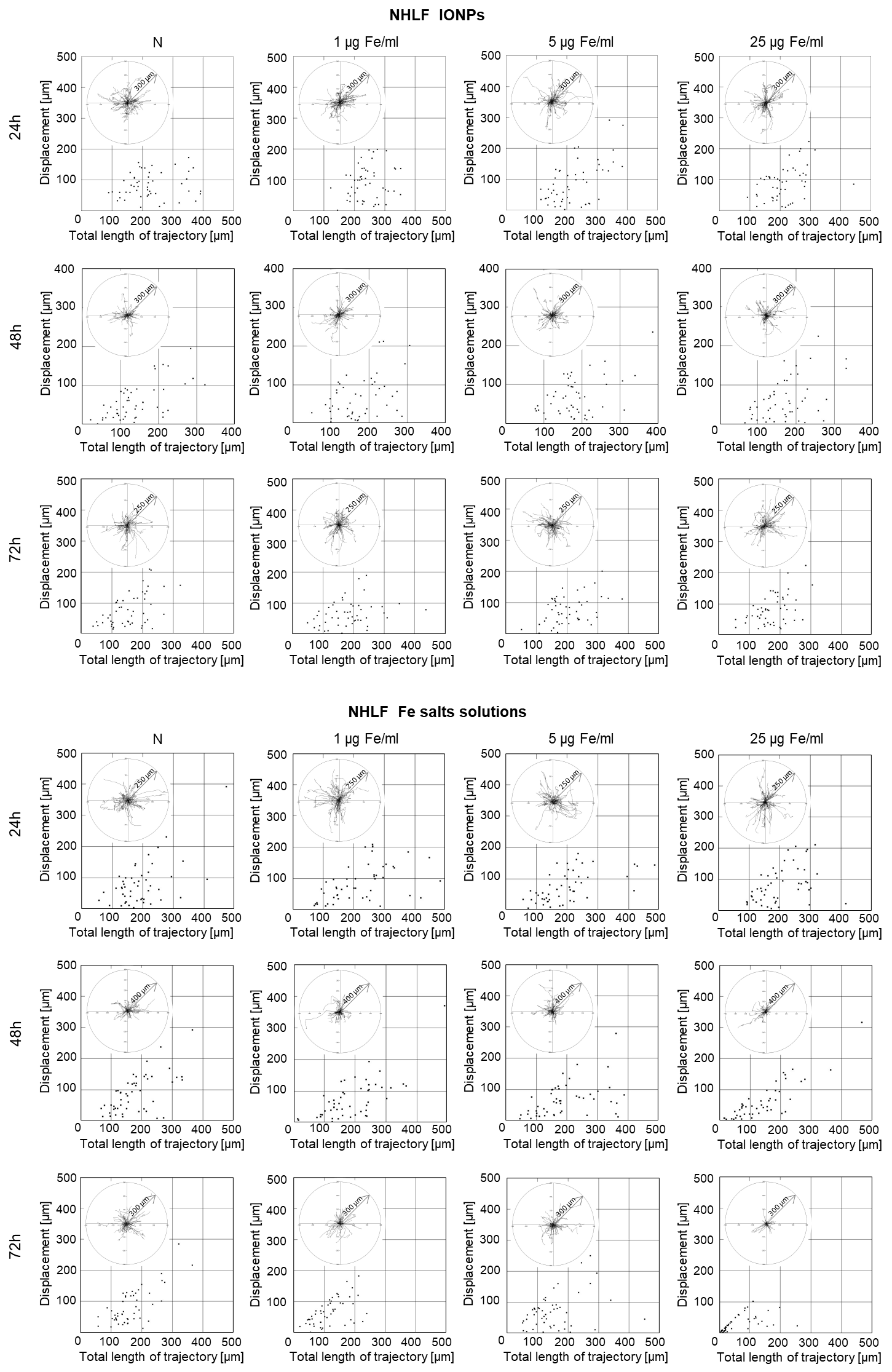

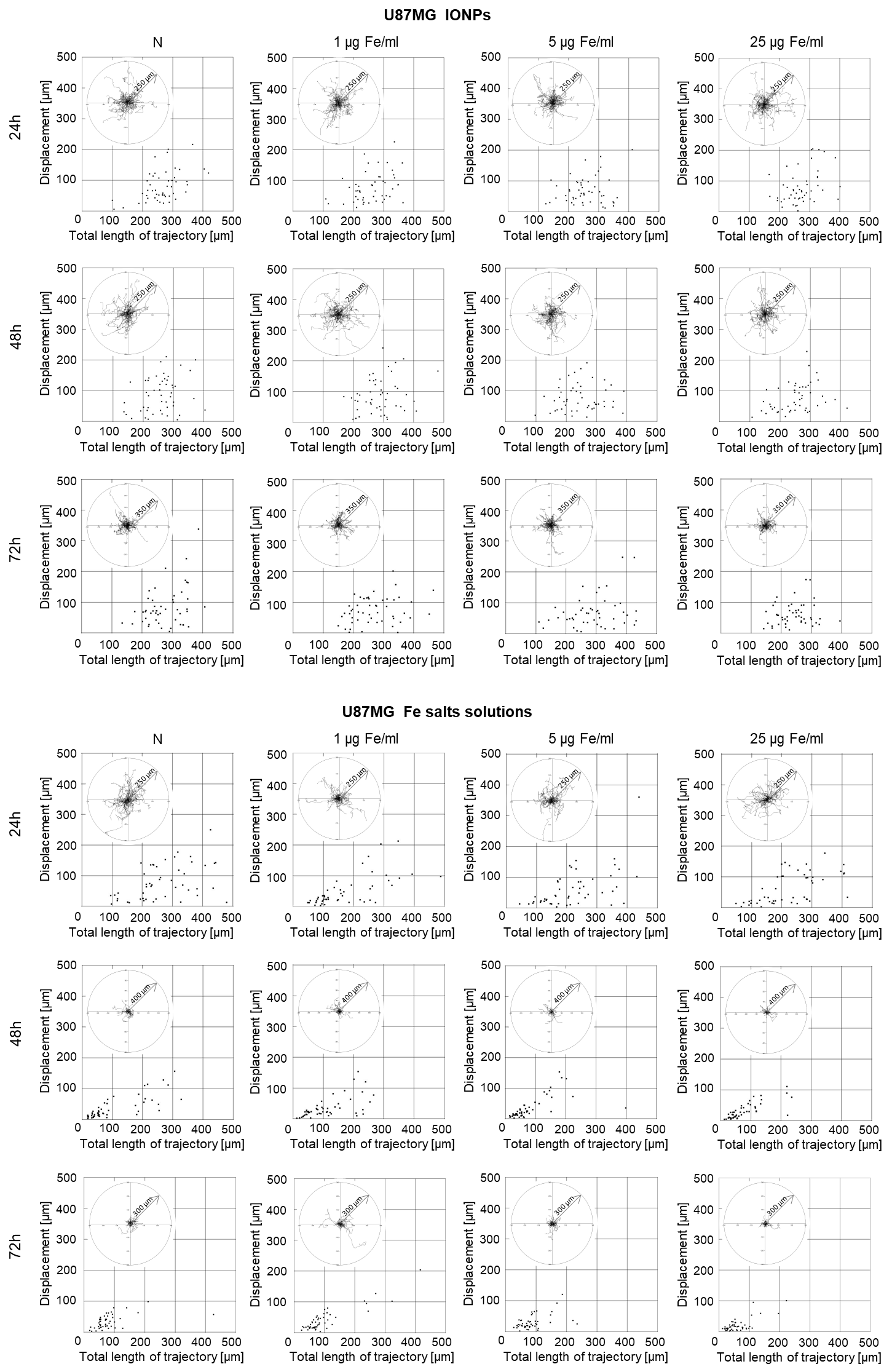

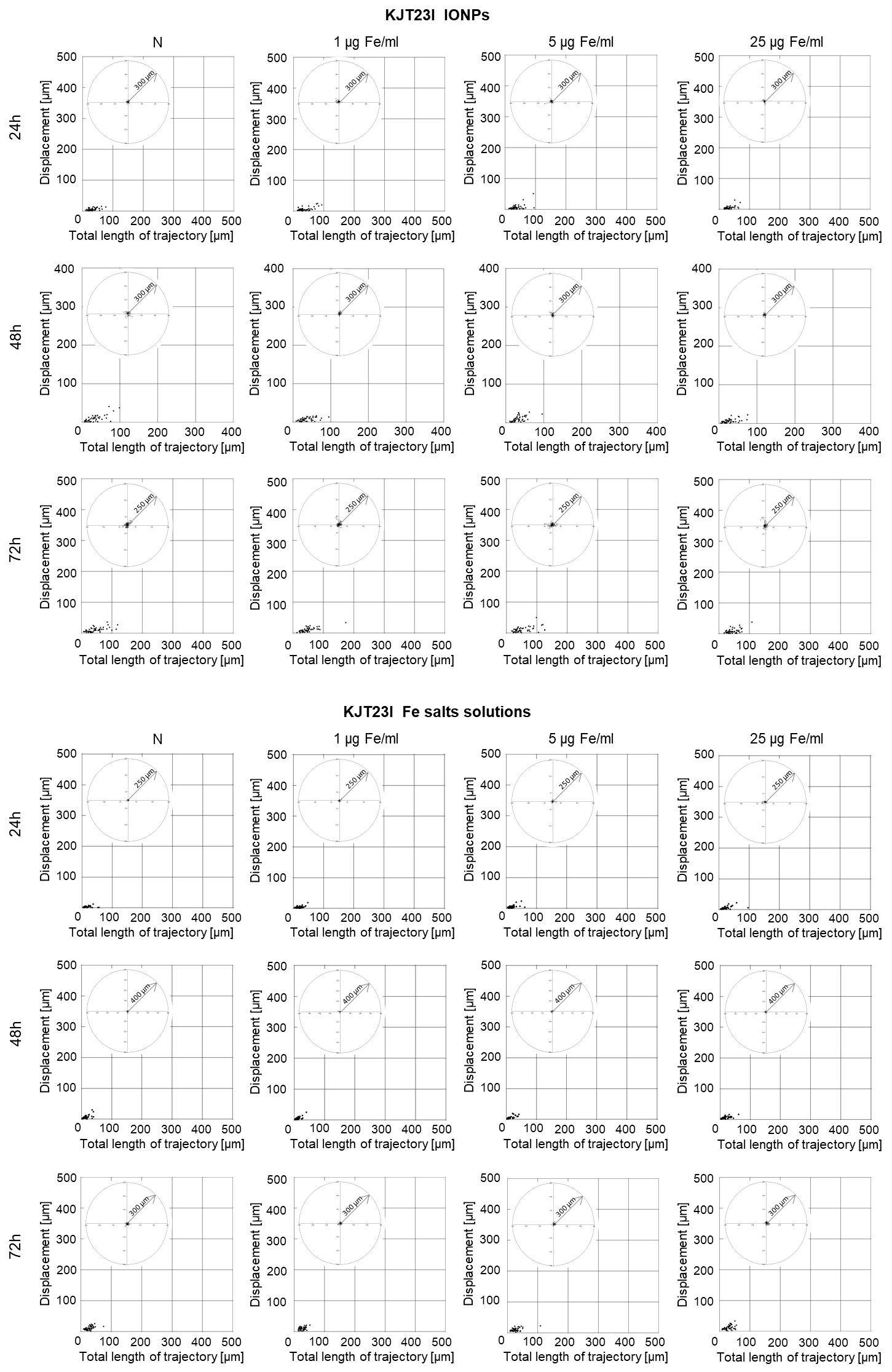


Figure 3S. Dot-plots depict displacement and total length of trajectory (distance) calculated for single macrophages exposed to IONPs and iron salts solutions as well as for corresponding control groups N. Circular plots present trajectories of individual cells.

Figure 4S. Dot-plots depict displacement and total length of trajectory (distance) calculated for single NHLF cells exposed to IONPs and iron salts solutions as well as for corresponding control groups N. Circular plots present trajectories of individual cells.

Figure 5S. Dot-plots depict displacement and total length of trajectory (distance) calculated for single U87MG cells exposed to IONPs and iron salts solutions as well as for corresponding control groups N. Circular plots present trajectories of individual cells.

Figure 6S. Dot-plots depict displacement and total length of trajectory (distance) calculated for single KJT23I cells exposed to IONPs and iron salts solutions as well as for corresponding control groups N. Circular plots present trajectories of individual cells.


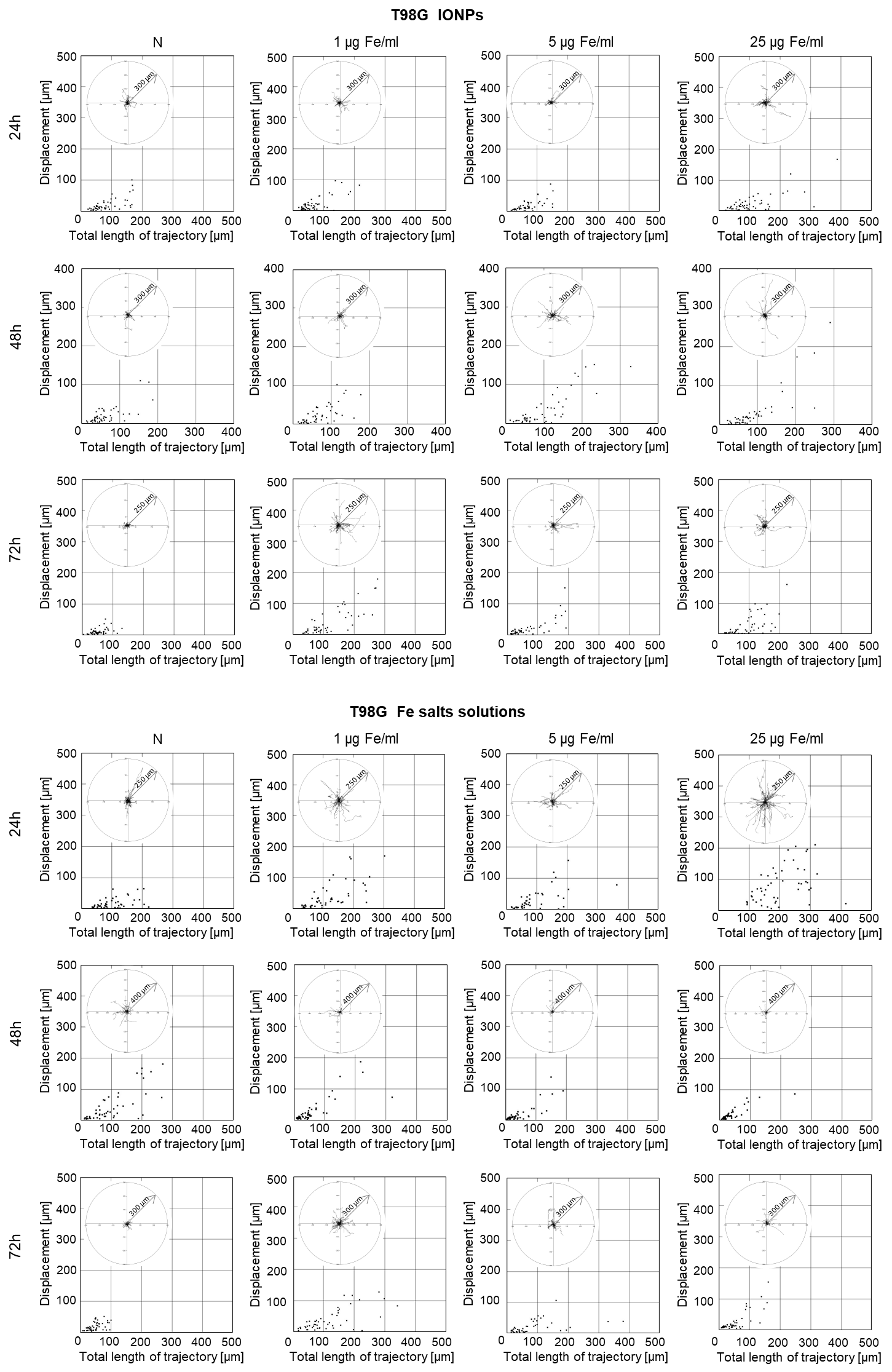


Figure 7S. Dot-plots depict displacement and total length of trajectory (distance) calculated for single T98G cells exposed to IONPs and iron salts solutions as well as for corresponding control groups N. Circular plots present trajectories of individual cells.
